# The Restriction Activity Investigation of Rv2528c, an Mrr-like Modification-Dependent Restriction Endonuclease from *Mycobacterium tuberculosis*

**DOI:** 10.3390/microorganisms12071456

**Published:** 2024-07-18

**Authors:** Tong Liu, Wei Wei, Mingyan Xu, Qi Ren, Meikun Liu, Xuemei Pan, Fumin Feng, Tiesheng Han, Lixia Gou

**Affiliations:** 1 Hebei Province Key Laboratory of Occupational Health and Safety for Coal Industry, School of Public Health, North China University of Science and Technology, Tangshan 063210, China; liu_tt1206@163.com (T.L.); xumingyan0518@163.com (M.X.); renqi@ncst.edu.cn (Q.R.); liumeikun2019@126.com (M.L.); 18861999241@163.com (X.P.); fm_feng@sina.com (F.F.); 2 Centers for Disease Control and Prevention of He Xi District, Tianjin 300210, China; wei_wei96@163.com; 3 School of Life Science, North China University of Science and Technology, Tangshan 063210, China

**Keywords:** Rv2528c, Mrr, *Mycobacterium tuberculosis*, restriction–modification system, type IV restriction endonuclease

## Abstract

*Mycobacterium tuberculosis* (*Mtb*), as a typical intracellular pathogen, possesses several putative restriction–modification (R-M) systems, which restrict exogenous DNA’s entry, such as bacterial phage infection. Here, we investigate Rv2528c, a putative Mrr-like type IV restriction endonuclease (REase) from *Mtb* H37Rv, which is predicted to degrade methylated DNA that contains m6A, m5C, etc. Rv2528c shows significant cytotoxicity after being expressed in *Escherichia coli* BL21(DE3)pLysS strain. The Terminal deoxynucleotidyl transferase dUTP Nick-End Labeling (TUNEL) assay indicates that Rv2528c cleaves genomic DNA in vivo. The plasmid transformation efficiency of BL21(DE3)pLysS strain harboring Rv2528c gene was obviously decreased after plasmids were in vitro methylated by commercial DNA methyltransferases such as M.EcoGII, M.HhaI, etc. These results are consistent with the characteristics of type IV REases. The in vitro DNA cleavage condition and the consensus cleavage/recognition site of Rv2528c still remain unclear, similar to that of most Mrr-family proteins. The possible reasons mentioned above and the potential role of Rv2528c for *Mtb* were discussed.

## 1. Introduction

Tuberculosis (TB) is an ancestral communicable disease spreading worldwide that is the leading cause of death from a single infectious agent, ranking above HIV/AIDS. In 2020, about 1.5 million people died of TB [1]. *Mycobacterium tuberculosis* (*Mtb*) is the pathogen of TB with which about one quarter of people worldwide are infected. *Mtb* is a canonical intracellular parasitic bacterium. Human pulmonary alveolar macrophages (AMs) are the primary target of *Mtb*, which was phagocytized by AMs before residence and grown within it [2,3]. Surprisingly, as a intracellular parasite, *Mtb* possesses several putative restriction–modification (R-M) system genes (shown in Table 1) [4], which are generally responsible for defense against phage infection or foreign DNA entry.

R-M systems, which are widespread among bacteria, generally consist of two parts: a sequence-specific restriction endonuclease (REase) and a cognate DNA methyltransferase (MTase) that methylate the same recognition sites to protect the host DNA from cleavage [5]. Foreign DNAs lacking host-specific methylation patterns are degraded by REase after entering host cells. In some cases, the R-M system lacks the cognate REase, and the orphan MTase is believed to probably participate in prokaryotic epigenetic modification and regulation [6,7,8,9,10,11].

R-M systems could be divided into four basic types according to the coupling relationship between REase and MTase, the type of recognition site, the distance between recognition site and the cleavage site, whether cofactors are needed, etc. [5]. Among them, type IV systems are often composed of one or two REase proteins that cleave only modified foreign DNA, including methylated, hydroxy-methylated, or glucosyl-hydroxy-methylated adenine or cytosine bases. Type IV REases show weak specificity; hence, their precise recognition sites are usually not well defined.

Mrr (Methylated adenine recognition and restriction) protein from *Escherichia coli* K-12 strains (EcoKMrr) is one of the mostly investigated type IV REases, which restricts DNAs containing N6-methyladenine (m6A) or C5-methylcytosine (m5C) in vivo [12,13]. The accurate recognition/cleavage sites or in vitro cleavage properties of EcoKMrr are not clear yet [13,14], while its in vivo genotoxicity was observed when genes of adenine or cytosine MTases (M.TaqI or M.HhaII, etc.) were expressed in recombinant *E. coli* strains harboring *mrr* gene [13,15], causing genomic DNA double-strand breaks and the subsequent SOS response for DNA repair [12]. Recent studies show that the in vivo activity of EcoKMrr can also be triggered by high hydrostatic pressure (HP) [16,17,18,19].

**Table 1:** R-M system proteins of *Mycobacterium tuberculosis* H37Rv.

Protein	Feature	Function
Rv0058	PI-MtuHIP	putative intein homing REase
Rv1461	PI-MtuHIIP	putative intein homing REase
Rv2024c	MamB	MTase [9]
Rv2528c	Mrr	putative Type IV REase
Rv2737c	PI-MtuI	intein homing REase [20,21,22]
Rv2755c	HsdS.1	Type I specificity subunit (inactive) [9]
Rv2756c	M.MtuHI	Type I MTase [8,9,10,11]
Rv2761c	HsdS	Type I specificity subunit [9]
Rv2966c		secretory MTase [23,24]
Rv3263	M.MtuHIII (MamA)	MTase [8]

**Figure 1:**
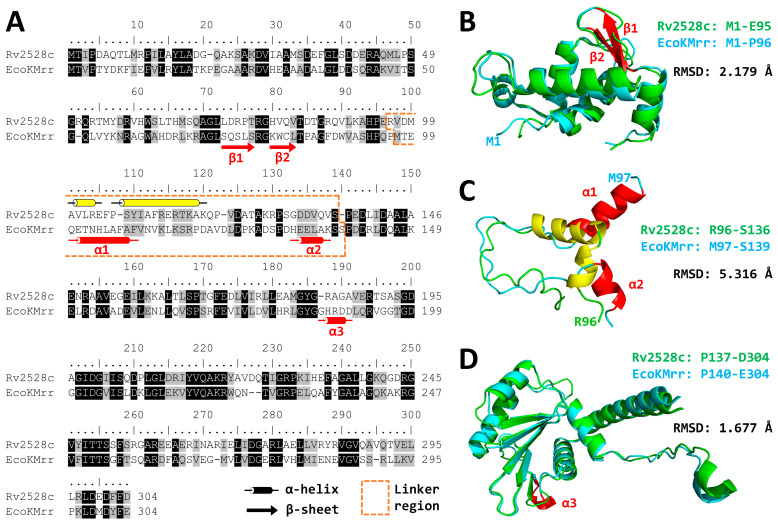
Structure comparison between Rv2528c and EcoKMrr. (**A**) Amino acid sequence alignment. Arrows denote β-sheets, cylinders denote α-helixes, and the dotted box in orange color circles the “linker” region for both proteins. The right panel shows the predicted tertiary structure superposition of the N-terminal region (**B**), the “linker” region (**C**), and C-terminal region (**D**) for both proteins, where the α-helixes and β-sheets are corresponding with (**A**). All the red-colored secondary structures are from EcoKMrr and yellow structures are from Rv2528c. Both tertiary structures are obtained from the AlphaFold database; the corresponding Uniprot ID for Rv2528c is I6Y9K2, and for EcoKMrr it is P24202.

Among *Mtb* R-M system proteins (Table 1), Rv2528c is the only hypothetical REase, except for three intein homing endonucleases that were not responsible for the defense mechanism [25]. Rv2528c shows significant identities with EcoKMrr (Figure 1A), hinting that they have similar functionalities to each other.

In this research, we compared the protein structure of Rv2528c with EcoKMrr, characterized the genotoxicity and DNA damage phenomenon of Rv2528c in *E. coli* BL21(DE3)pLysS strains, and investigated the DNA modification types recognized by Rv2528c in vivo. Finally, the possible role of Rv2528c and the potential of this research for improving the efficacy of phage therapy towards TB were discussed.

## 2. Materials and Methods

### 2.1. Strains, Enzymes and Plasmid Constructions

The commonly used *E. coli* strains JM110, Top10, and BL21(DE3)pLysS were stored in a laboratory previously. The commercial MTases listed in Table 2 were all purchased from New England Biolabs, Hitchin, UK. The total Rv2528c coding sequence without stop codon was obtained from the Mycobrowser database (https://mycobrowser.epfl.ch), accessed on 12 October 2022 [26] and was commercially synthesized and cloned to the pET44b vector between NdeI and XhoI sites (GenScript Corporation, Zhenjiang, China), named Rv2528c-pET44bΔ.

Since the intact pET44b contains an extra 1823 bp sequence between NdeI and XhoI sites, so as to generate a cognate blank control plasmid, the 1823 bp sequence was substituted with two stop codons (TAATAG), named pET44bΔ. This construction was carried out firstly via PCR, using pET-F: 5′-CTATTACAATGTATATCTCCTTCTTAAAG-3′ and pET-R: 5′-CTCGAGCACCACCACCACCACCAC-3′ as primers to amplify the pET44b plasmid. Then, the PCR product was purified using the gel recovery method (D2500, Omega Bio-tek, Norcross, GA, USA) prior to self-ligation via T4 PNK and T4 ligase (New England Biolabs, Beverly, MA, USA) under 16 °C for 1h. The ligation product was transformed into Top10 competent cells, followed by screening and validation via sequencing.

### 2.2. Sequence Alignment and Tertiary Structure Superposition

The amino acid sequences of Rv2528c and EcoKMrr (GenBank: AAC77307.1) were aligned using ClustalW built in BioEdit 7.1 software. The PDB files of both proteins (I6Y9K2 and P24202) were downloaded from the AlphaFold Protein Structure Database (https://alphafold.ebi.ac.uk/), accessed on 12 May 2022 [28]. The tertiary structure analysis and superposition comparison were all performed using PyMOL 2.6.

The Root Mean Squares Deviation (RMSD) value of each superposition was acquired using the “align” command with the number of parameter cycles = 0 to compare all the atoms.

### 2.3. Competent Cell Preparation and Electroporation Transformation

**Figure 2:**
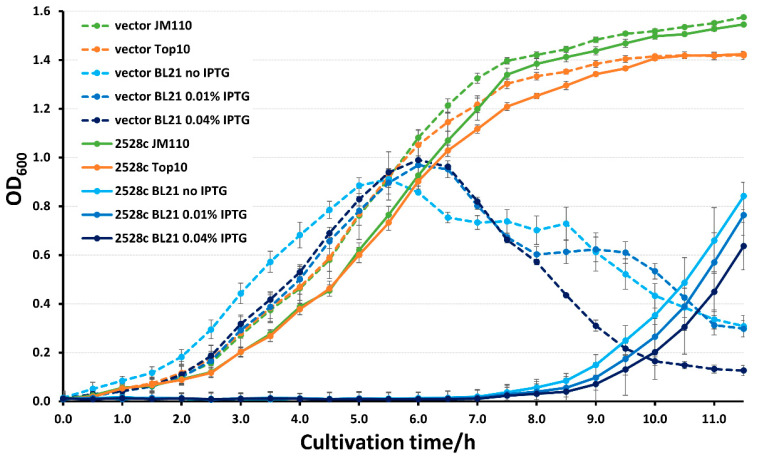
Growth curve of JM110, Top10, and BL21 strains harboring Rv2528c-pET44bΔ or the blank pET44bΔ plasmid. A final concentration of 0.01% or 0.04% IPTG (0.8 M) was added into the culture medium to induce relatively low or moderate expression of Rv2528c. Strains containing blank pET44bΔ vector were dot-lined, and the corresponding Rv2528c-pET44bΔ-containing strains were solid-lined with the same color.

The inoculation and incubation of *E. coli* strains, as well as CaCl_2_ competent cell and electroporation competent cell preparation, were performed following the standard procedure descried in Molecular Cloning [29]. Due to the genotoxicity of Rv2528c, the BL21/Rv2528c-pET44bΔ electroporation competent cells should be freshly prepared, and electroporation transformation should be carried out immediately. The electroporation competent cells were freshly prepared just before transformation due to the weak growth of BL21/Rv2528c-pET44bΔ strain (Figure 2), which even makes the efficiency of the CaCl_2_ transformation method too low to use.

### 2.4. Growth Curve Measurement

The JM110, Top10, and BL21(DE3)pLysS strains harboring Rv2528c-pET44bΔ or pET44bΔ, respectively, were cultured overnight and then inoculated into a sterilized 96-well microplate. Briefly, 200 μL LB medium was added per well with respective antibiotics, and then the overnight culture was added and adjusted to OD_600_ = 0.01. Each sample was replicated for three wells. Furthermore, BL21/Rv2528c-pET44bΔ and BL21/pET44bΔ strains were additionally supplied with a final concentration of 0.01% or 0.04% IPTG (0.8 M), respectively, to induce a relatively low or moderate expression of Rv2528c.

Then, the 96-well microplate was covered with a sterilized transparent lid and incubated in a shaker under 37 °C and 220 rpm shaking. The OD_600_ value of each well was measured with a microplate reader (SpectraMax M5, Molecular Devices Company, San Jose, CA, USA) every 30 min.

### 2.5. Western Blot Detection for In Vivo Recombinant Rv2528c Expression

The BL21 cell cultures were collected and lysed with RIPA lysis buffer (Thermo Fisher Science, Waltham, MA, USA), and the sample protein concentration was quantified with a BCA kit (Thermo Fisher Science). Then, 12% separation gel was disposed for SDS-PAGE. After transfer, 5% skim milk powder was blocked at room temperature for 2 h prior to wash. Then, the PVDF membrane were incubated overnight at 4 °C with 6*His-Tag MouseMonoclonal antibody. The secondary antibody (Goat Anti-Mouse IgG H&L (HRP) Preadsorbed ab97040) was incubated for 2 h at room temperature and developed after rinsing the next day. Tri-replicates were examined for each sample.

### 2.6. In Vivo DNA Cleavage Assay (TUNEL Kit) and Flow Cytometry Detection

The overnight cultures of BL21/pET44bΔ (one sample) and BL21/Rv2528c-pET44bΔ (triplicate samples) were inoculated into LB medium containing ampicillin (100 μg/mL) and chloramphenicol (25 μg/mL) in a ratio of 1:50 and incubated for 3–4 h to OD_600_ = 0.6. Afterward, two samples of BL21/Rv2528c-pET44bΔ were added to a final concentration of 0.01% or 0.04% IPTG (0.8 M), respectively, and incubated for another 1 h.

Next, all four samples were collected and resuspended in PBS solution and detected with the TUNEL kit following the manufacturer’s guide (C1086, Beyotime company, Haimen, China). The final FITC-dUTP-labeled products were resuspended in PBS and detected by flow cytometry (BD Accuri C6).

For each sample, 100,000 events were collected, and the threshold was set to FSC-H 36,000 and SSC-H 3600. The frequency distribution plot for FL1-A channel (488 nm → 533/30 nm) was monitored and recorded and then analyzed with CFlow Plus 1.0.264.15

### 2.7. In Vitro Modification of pCDFDuet and pRSFDuet

The pRSFDuet plasmid was firstly isolated from JM110 and Top10 strains following the SDS-alkaline lysis minipreparation method [29]. Then, plasmids purified from JM110 were modified in vitro by commercial MTases (New England Biolabs, UK) (Table 1) according to the manufacturer’s instructions. The modified products were recovered and concentrated using a gel extraction kit (D2500, Omega Bio-tek), followed by electroporation transformation into the freshly prepared BL21/Rv2528c-pET44bΔ or BL21/pET44bΔ competent cells. The transformants’ CFU numbers were normalized against the amount of DNA used per treatment.

### 2.8. Protein Expression, Purification, and In Vitro Cleavage Condition Explorations

A 10 mL overnight culture of BL21/Rv2528c-pET44bΔ was inoculated into 1000 mL LB medium supplied with 100 μg/mL ampicillin and 25 μg/mL chloramphenicol and was grown at 37 °C to OD_600_ = 0.6. Then, it was cooled to room temperature and a final concentration of 0.4 mM IPTG was added, followed by further incubation for 5 h at 25 °C. The purification procedure was accomplished as described in [30] with a minor difference in that the linear gradient of imidazole was from 0 to 1000 mM.

The in vitro DNA cleavage condition explorations were performed following the similar strategy and protocol described in [31], except for that the DNA substrates were pRSFDuet or other large plasmids modified by commercial MTases, and the tested protein was C-terminal 6xHis-tagged Rv2528c. The commercial restriction buffer CutSmart (New England Biolabs) was also tested and employed as a cleavage buffer along with NEB buffers 3.1 and 2.1.

## 3. Results

### 3.1. Protein Structures of Rv2528c Compared with EcoKMrr

As shown in Figure 1, the amino acid sequence of Rv2528c shares 36% identities and 57% similarities to that of EcoKMrr. For the sake of further comparison, the protein tertiary structure superposition was necessary. Because there were no crystal structure data for both proteins in the PDB database [32], we adopted the predicted three-dimensional structures of Rv2528c and EcoKMrr that were pre-calculated in the AlphaFold Protein Structure Database [28].

To quantitatively compare the structural similarities between Rv2528c and EcoKMrr, the Root Mean Squares Deviation (RMSD) was acquired after superposition comparison. RMSD measures the minimal average distance (Angstrom as unit) of corresponding atoms between two proteins after superposition, which means that the smaller the RMSD value is, the more similar the two proteins are.

Unexpectedly, the full structure superposition between Rv2528c and EcoKMrr showed poor structural similarity (RMSD = 20.153 Å, Figure S1C). But substructure comparison, such as the N-terminal region of Rv2528c from residues M1 to E95 and M1 to P96 for EcoKMrr (RMSD = 2.179 Å, Figure 1B), or the C-terminal region of Rv2528c from residues P137 to D304 and P140 to E304 for EcoKMrr (RMSD = 1.677 Å, Figure 1D), exhibits fairly high coincidence. The slight inconsistence among these two regions was two short β-sheets (in red color) in the N-terminal region (Figure 1A,B) and one shorter α-helix (in red color) in the C-terminal region (Figure 1A,D), which are all located in EcoKMrr.

The main difference comes from the “linker” region (dotted box in orange color), which is residues R96 to S136 for Rv2528c and residues M97 to S139 for EcoKMrr (RMSD = 5.316 Å, Figure 1A). The superposition results revealed that there were two α-helixes (in yellow color) from Rv2528c that were translocated in comparison with that of EcoKMrr (in red color) (Figure 1A,C). This translocation, which makes the two α-helixes (in yellow color) closer to each other, may increases the rigidity of the “linker” region, resulting in a further distance between the N- and C-terminal domain of Rv2528c compared with that of EcoKMrr (Figure S1A,B). The structural and functional changes in Rv2528c influenced by this translocation are pending further investigation.

### 3.2. In Vivo Genotoxicity of Rv2528c in E. coli BL21(DE3)pLysS Strain

While incubating the expression strain BL21 (DE3) pLysS containing Rv2528c-pET44bΔ, obvious growth retardation was observed even without adding IPTG into the LB medium. The most probable reason for this was the background expression of Rv2528c, either because of its (over) expression or the possible type IV REase genotoxicity, since BL21 strains possess Dam DNA methylation modification (5′-GATC-3′) which means that the genomic DNA could be recognized and cleaved by Rv2528c.

Then, the growth curve of BL21 (DE3) pLysS strain containing Rv2528c-pET44bΔ was determined to validate this retardation phenotype in parallel with two commonly used cloning strains, Top10 and JM110, as positive controls. Top10 and JM110 lack the DE3 element that is indispensable for coding sequence expression via pET series vectors; hence, Rv2528c would have no background expression in these two strains. The blank pET44bΔ vector was also introduced into these three strains as the negative control. The background expression of Rv2528c in BL21 strain (without IPTG induction) was detected by Western blot, using 6xHis-tag mouse monoclonal antibody as the primary antibody. As Figure S2 showed, there was no obvious expression of 6xHis-tagged Rv2528c in vivo compared with that of 0.01% or 0.04% IPTG induction.

From Figure 2, we can conclude that the background-, low-, and moderate-level expression of Rv2528c in BL21 (DE3) pLysS strains leads to significant bacterial growth retardation, while JM110 or Top10 harboring Rv2528c-pET44bΔ grows normally owing to the absence of the DE3 element of the host.

It is unusual that the growth curves for BL21 harboring blank pET44bΔ vectors were gradually decreased after the late-exponential phase even without a stationary phase. This phenomenon may be due to the metabolic burden introduced by the pET expression system for BL21, or an alternative self-curing mechanism where the BL21 strains eliminate the pET44bΔ plasmid with a longer cultivation period and, furthermore, the hypoxic environment caused by lid-covering and insufficient shaking of the microplate. In Rv2528c expressing BL21 strains, the toxicity of Rv2528c may exacerbate plasmid elimination, causing delayed growth. This mechanism can also explain the poor growth of BL21 strains with no-IPTG induction (Figure 2) since Rv2528c was barely expressed (Figure S2).

To check whether this growth retardation was caused by the in vivo cleavage of genomic DNA by Rv2528c, the Terminal deoxynucleotidyl transferase dUTP Nick-End Labeling (TUNEL) assay was performed for the late-exponential phase cultures of BL21(DE3)pLysS strains obtaining Rv2528c-pET44bΔ (abbreviated as BL21/Rv2528c-pET44bΔ) or pET44bΔ blank vector (abbreviated as BL21/pET44bΔ). The TUNEL assay was routinely used to monitor cell apoptosis caused by DNA double-strand breaks in vivo for both eukaryotic and prokaryotic cells, in which the 3′-OH of DNA breaks were fluorescently labeled with FITC-dUTP by TdT enzyme [33]. Cells stained via TUNEL were subsequently analyzed by flow cytometry (BD Accuri C6).

**Figure 3:**
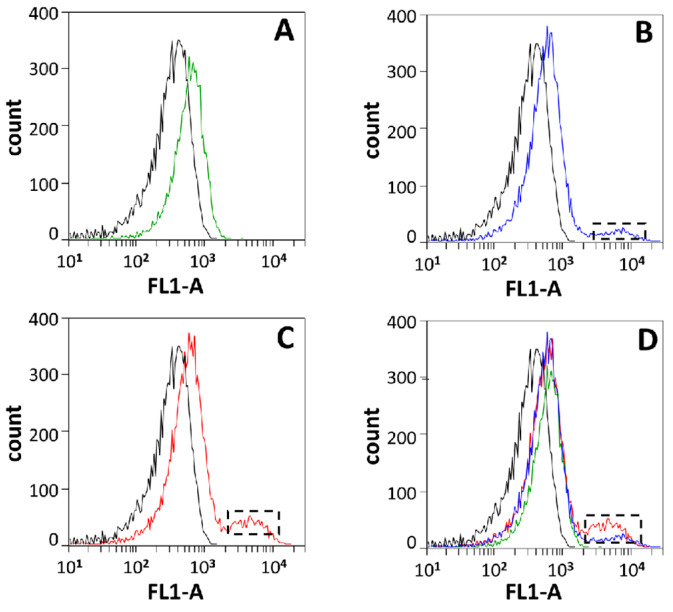
Fluorescence frequency distribution diagram of TUNEL-stained strains. The late-exponential phase cultures of BL21/Rv2528c-pET44bΔ without IPTG inducing (**A**), with 0.01% IPTG (0.8 M) (**B**) or 0.04% IPTG (0.8 M) inducing (**C**), as well as their overlap plot (**D**), all detected by flow cytometry. The black curve in each plot represents the negative control that is BL21/pET44bΔ without IPTG inducing. FL1-A: the area of green fluorescence (488 nm → 533/30 nm).

As Figure 3 shows, in contrast with the negative control (black curve denotes the BL21/pET44bΔ strain without IPTG inducing, mean value = 337.94), the background-level expression of Rv2528c leads to obvious DNA damage (Figure 3A, green curve, mean value = 630.62), and the damage was aggravated after the Rv2528c expression level was raised by adding a final concentration of 0.01% or 0.04% IPTG (0.8 M) to the culture (the black-dotted box in Figure 3B,C and blue and red curve, mean values = 970.36 and 1133.13, respectively). Figure 3D shows the merged image for Figure 3A–C. These results indicate that Rv2528c may cleave genomic DNA in vivo, consistent with the characteristics of Mrr-like REases.

### 3.3. In Vitro Cleavage Assay of Rv2528c

**Figure 4:**
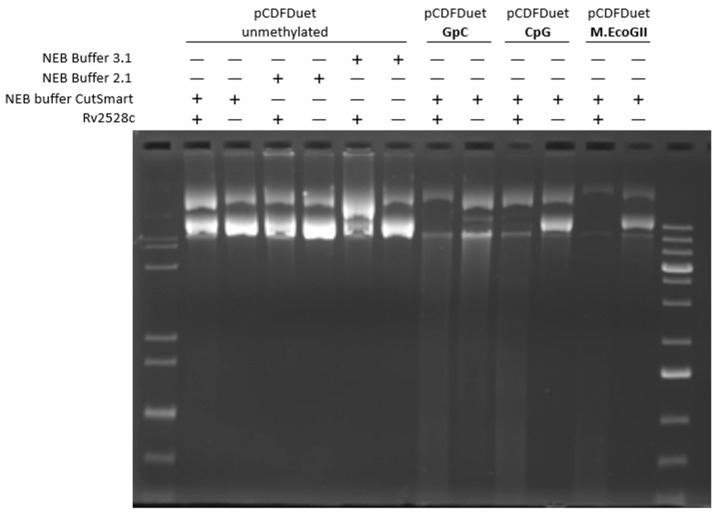
In vitro cleavage assay of Rv2528c on modified or non-modified pCDFDuet plasmid DNA. Boiled Rv2528c was used as negative control.

To investigate the in vitro cleavage activity of Rv2528c, the C-terminal 6xHis-tagged Rv2528c protein was successfully purified (Figure S3). The supercoiled plasmid DNA substrate pCDFDuet was firstly modified by DNA MTase GpC, CpG, and M.EcoGII. The modification sites of these three MTases are short enough to cover the vast majority of cytosine or adenine modifications. As shown in Figure 4, Rv2528c showed non-specific degrading activity on these three modified DNA, and unmethylated DNA was not cleaved.

Unfortunately, further attempts to investigate the cleavage of Rv2528c on DNA substrates modified by other DNA MTases such as M.HpaII, M.HhaI, and M.MspI (see list in Table 2) failed. These results were consistent with EcoKMrr and most of its homologs in terms of the fact that they can recognize and restrict lots of DNA modification types in vivo, but few specific cleavage activities in vitro were observed [13,14,15].

### 3.4. Investigation of DNA Methylation Type Recognized by Rv2528c In Vivo

To fully survey the DNA methylation types that Rv2528c recognizes, a series of commercial DNA MTases (Table 2) were employed to modify a test plasmid pRSFDuet in vitro. The specific recognition site numbers on pRSFDuet for each MTase used here are listed in Table 2. pRSFDuet carries a RSF replication origin and a kanamycin-resistant gene that were both different from that of pET44bΔ; therefore, these two plasmids were compatible in the same host [34]. Before being transformed into BL21/Rv2528c-pET44bΔ, the pRSFDuet plasmid was modified in vitro by each commercial MTase, respectively, following the manufacturer’s protocols.

Additionally, pRSFDuet plasmid was also isolated from JM110 or Top10 strain, respectively. It is well known that *E.coli* contains three types of DNA MTases, HsdM that recognizes 5′-AACNNNNNNGTGC-3′ [35,36], Dam that recognizes 5′-GATC-3′, and Dcm that recognizes 5′-CCWGG-3′(N = G/A/T/C, W = A/T), while underlined bases denote the methylation site [36]. The DNA methylation-related genotype of JM110 is hsdM+, dam−, and dcm−, and Top10 is hsdM−, dam+, and dcm+. pRSFDuet lacks the HsdM recognition site (see Table 2), so this plasmid purified from JM110 carries no DNA methylation (as positive control), while pRSFDuet from Top10 strain contains Dam and Dcm methylation.

All 13 types of modified pRSFDuet plasmids were transformed into BL21/Rv2528c-pET44bΔ or BL21/pET44bΔ, respectively, via electroporation, without IPTG inducing.

**Figure 5:**
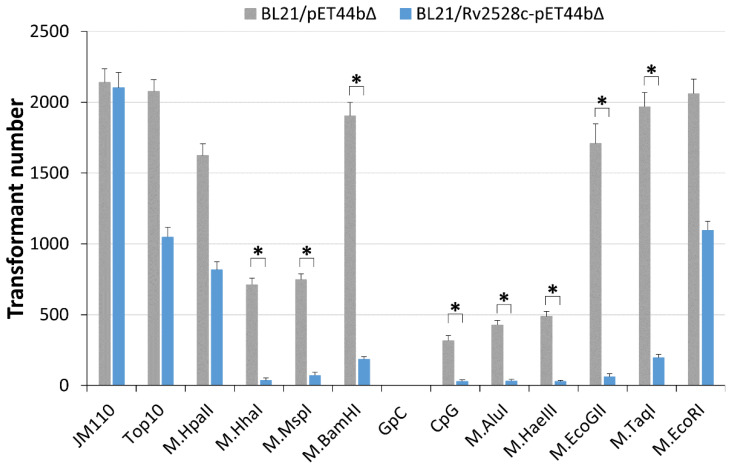
Transformation efficiency comparison of pRSFDuet plasmids modified by commercial MTases. All plasmids were electroporated into BL21/Rv2528c-pET44bΔ or BL21/pET44bΔ strains. pRSFDuet isolated from JM110 strain (without any DNA methylation, as the positive control) or Top10 strain (dam+, dcm+) was also tested. The * denotes more than 10-fold decrease in the transformation efficiency.

It is noteworthy that if the plasmid was modified by GpC/CpG/M.AluI or M.HaeIII, its transformation efficiency against BL21/pET44bΔ (the blank control host) was also decreased obviously, regardless of Rv2528c existence. This restriction phenotype was owing to a newly discovered type IV REase in *E. coli* B lineage, named EcoBLI [37] (or EcoMcrX [38,39]). All BL21 strains contain this enzyme, which recognizes and cleaves the 5′-GCNGC-3′ (N = G/A/T/C) pattern [37], or a more relaxed but less specific 5′-RCSRC-3′ (R = G/A, S = G/C) motif [38,39]. The recognition site of GpC/CpG/M.AluI or M.HaeIII shares overlap with that of EcoBLI (Table 2); thus, it could obviously be restricted. Among them, GpC possesses the greatest number of overlaps, which makes it completely lethal to the BL21 host (Figure 5). The relative low efficiency for that of M.HhaI and M.MspI may also be contributed by the non-specific cleavage of EcoBLI [38].

From Figure 5, we can find that the transformation efficiency for DNA carrying M.HhaI, M.MspI, M.BamHI, CpG, M.AluI, M.HaeIII, M.EcoGII, and M.TaqI against BL21/Rv2528c-pET44bΔ was significantly decreased more than 10-fold (marked as *) compared to that of BL21/pET44bΔ. These modification types may be the targets of Rv2528c in vivo, even if taking the influence of EcoBLI into account. On the other hand, plasmid modified by M.HpaII or M.EcoRI or isolated from Top10 (carrying Dam and Dcm modification) showed less restriction by Rv2528c, for their decrease was less than two-fold. This preference may be related with the invasive foreign DNA modification types that *Mtb* encountered, which were then accumulated in the structure and function evolution of Rv2528c. The specific mechanism is pending further investigation.

The DNA modification types preferred by Rv2528c were almost the same as that of EcoKMrr [12,13], except for M.BamHI, a cytosine-N4 MTase that is different from the main cytosine-C5 MTases. This difference may come from the conformational difference between Rv2528c and EcoKMrr (Figure 1), or more generally, the DNA methylation circumstance difference in vivo and in vitro between *Mtb* and *E. coli*. Meanwhile, the precise recognition site of Rv2528c cannot be summarized clearly based on current results, just like EcoKMrr and most Mrr family REases.

## 4. Discussion

### 4.1. Difficulties for Determination of In Vitro Cleavage Activity of Rv2528c

In this research, much effort was paid to exploring the in vitro cleavage conditions of Rv2528c. In addition, with the commercial REase buffers shown in Figure 4, manually optimized buffers varying in terms of pH (7.0, 8.0, 9.0), divalent metal ions (Mg^2+^, Mn^2+^, Ni^2+^, Zn^2+^), monovalent salt ion concentration (Na^+^ or K^+^ at 50, 100, 150, 200 mM), and cofactors (NTP, dNTP, SAM, etc.) were also tested, with no obvious cleavage observed. Hence, detection of the major restriction activity was taken in vivo, mainly adopting the TUNEL assay to detect the DNA double-strand break in the early stage of cell growth, such as exponential phase [17,40,41].

Only unique Mrr-like subfamily proteins with a share of less than 20% that identify as EcoKMrr, such as MspJI, AspBHI, etc., were characterized in vitro, whereas their recognition sequences are still highly degenerate [42,43,44]. So, it is possible that the bona fide recognition sites of EcoKMrr or Rv2528c are scarce, and that complete sites are only distributed on a genome scale, or a special DNA structure is needed, like a replication fork. For instance, EcoKMcrBC, another type IV REase in *E. coli*, recognizes R^m^CN_40–2000_R^m^C (R is A or G) sites [45,46] and can cleave DNA replication fork in vitro [47]. This hypothesis can explain why the restriction phenotype of EcoKMrr or Rv2528c has been observed only in vivo to date, as perhaps the total recognition site sequence contains part of the host genomic sequence needed for assistance; hence, new insights are required for the determination of the in vitro activity of EcoKMrr or RV2528c.

### 4.2. The Possible Role of Rv2528c

As mentioned above, currently, the consensus recognition sites for EcoKMrr remain unclear; there is no in vitro endonuclease activity for the recombinant EcoKMrr or most Mrr-like proteins. These atypical REase features have blurred their actual biological functions.

Typical IV restriction endonucleases, the main function of Mrr family proteins, are able to resist phage infection. Mycobacteriophage (such as D29 or TM4) invasion or other forms of foreign DNA transformation could happen to *Mtb* before it infects the human body; thus, it can introduce heterologous MTase genes into *Mtb*’s genome, which would establish abnormal epigenetic regulations inside *Mtb*. Therefore, the incompatibility with foreign MTases caused by Rv2528c could protect the host from harmful DNA modifications or regulations. However, considering that most of the life cycle of *Mtb* is in the human body, the chance of it being infected by phage is extremely low. Therefore, Mrr may have other functions that prevent it from being eliminated during evolution.

In 2005, Aertsen et al. [16,17] reported that high hydrostatic pressure (HP, ~100 MPa) stress can activate the Mrr restriction activity of *E. coli* itself, causing non-specific genomic DNA cleavage and the subsequent SOS response *per se*. Further studies clarified that the natural EcoKMrr was an inactive tetramer, while HP shock or foreign MTase expression can induce the tetramer to dissociate into the active dimer state [18,19]. Therefore, Rv2528c may also have a similar function that responds to HP shock or other extreme external stresses, where it then degrades its own DNA to enter the dormant state, or activates the SOS response, which should be further investigated.

On the other hand, R-M systems of *Mtb* seem to be disappearing. For instance, the type I R-M system (Rv2756c and Rv2761c) lacks the REase subunit, and Rv3263 (MamA) lacks the cognate REase too. In contrast, the Rv3263 homolog in *M. smegmatis*, MSMEG_3213, retains its cognate REase MSMEG_3214 [48]. A possible reason for this may be related to the adaptive evolution of *Mtb*. In the natural environment, *Mtb* usually exists in the air and can maintain pathogenicity for 8–10 days. After infecting the human body, such as the lungs, it is rapidly phagocytized by human AMs. Both circumstances make it hard for mycobacterial phages and foreign DNAs to invade *Mtb*, so these REases may be abandoned gradually. Eventually, Rv2528c that possesses a wide restriction range for methylated foreign DNA stuck to the post as the last barrier. This reinforces the significance of the further investigation of Rv2528c’s biological functions.

## 5. Conclusions

In this research, we focus on a putative type IV restriction endonuclease (REase) Rv2528c, which is the sole REase candidate of *Mycobacterium tuberculosis* H37Rv. Rv2528c shares significant sequence and structure similarities with Mrr protein of *E. coli* K-12 strain (EcoKMrr) at the N- and C-terminal region, and the flexible middle region is distinct. Growth curve determination shows that the expression of Rv2528c in *E. coli* BL21(DE3)pLysS strains causes obvious genotoxicity, and this phenotype was further demonstrated by the Terminal deoxynucleotidyl transferase dUTP Nick-End Labeling (TUNEL) assay, which revealed that Rv2528c can degrade genomic DNA in vivo. Since most Mrr-like REases did not exert in vitro cleavage activities, the in vivo restriction test of Rv2528c was examined, and its recognition preference for methylation type was similar to that of EcoKMrr. Finally, we discussed the possible reason for the difficulties in exploring the in vitro activity of Rv2528c and Mrr-like proteins. The potential biological functions of Rv2528c and the adaptive evolution tendency were also surmised.

## Figures and Tables

**Table 2:** MTases used and their recognition site numbers in pRSFDuet plasmid.

Strain/MTase	Modification Site	Site Number in pRSFDuet	Sites Overlapping with EcoBLI (GCNGC)
**Strains**			
JM110	AAC(N_6_)GTGC ^1,2^	0	0
Top10	GATC and CCWGG ^2^	10 and 9	0
**MTases**			
M.HpaII	CCGG	20	0
M.HhaI	GCGC	28	0
M.MspI	CCGG	20	0
M.BamHI ^3^	GGATCC	1	0
GpC	GC	288	27
CpG	CG	258	12
M.AluI	AGCT	17	3
M.HaeIII	GGCC	12	4
M.EcoGII	A	979	0
M.TaqI	TCGA	15	0
M.EcoRI	GAATTC	1	0

^1^ Underlined base denotes the methylation site, ^2^ N = A or T or G or C; W = A or T, ^3^ M.BamHI is a cytosine-N4 MTase that is different from the common cytosine-C5 MTases [27].

## Data Availability

The original contributions presented in the study are included in the article/Supplementary Material, further inquiries can be directed to the corresponding authors.

## Supplementary Materials

The following supporting information can be downloaded at: https://www.mdpi.com/article/10.3390/microorganisms12071456/s1, Figure S1: AlphaFold-predicted tertiary structure of Rv2528c and EcoKMrr, as well as their superposition; Figure S2: Western blot analysis for in vivo Rv2528c expression in *E. coli* BL21(DE3)/pLysS after IPTG induction; Figure S3: SDS-PAGE analysis for purification of the C-terminal 6xHis-tagged Rv2528c.
