# Supplementary material for: The Restriction Activity Investigation of Rv2528c, an Mrr-like Modification-Dependent Restriction Endonuclease from Mycobacterium tuberculosis
Source: Microorganisms. 2024 Jul 18;12(7):1456. doi: 10.3390/microorganisms12071456 (PMC11279042; doi:10.3390/microorganisms12071456)
Supplement: Supplementary file 1 [file microorganisms-12-01456-s001.zip › microorganisms-3071136-supplementary.pptx]

## Slide 1
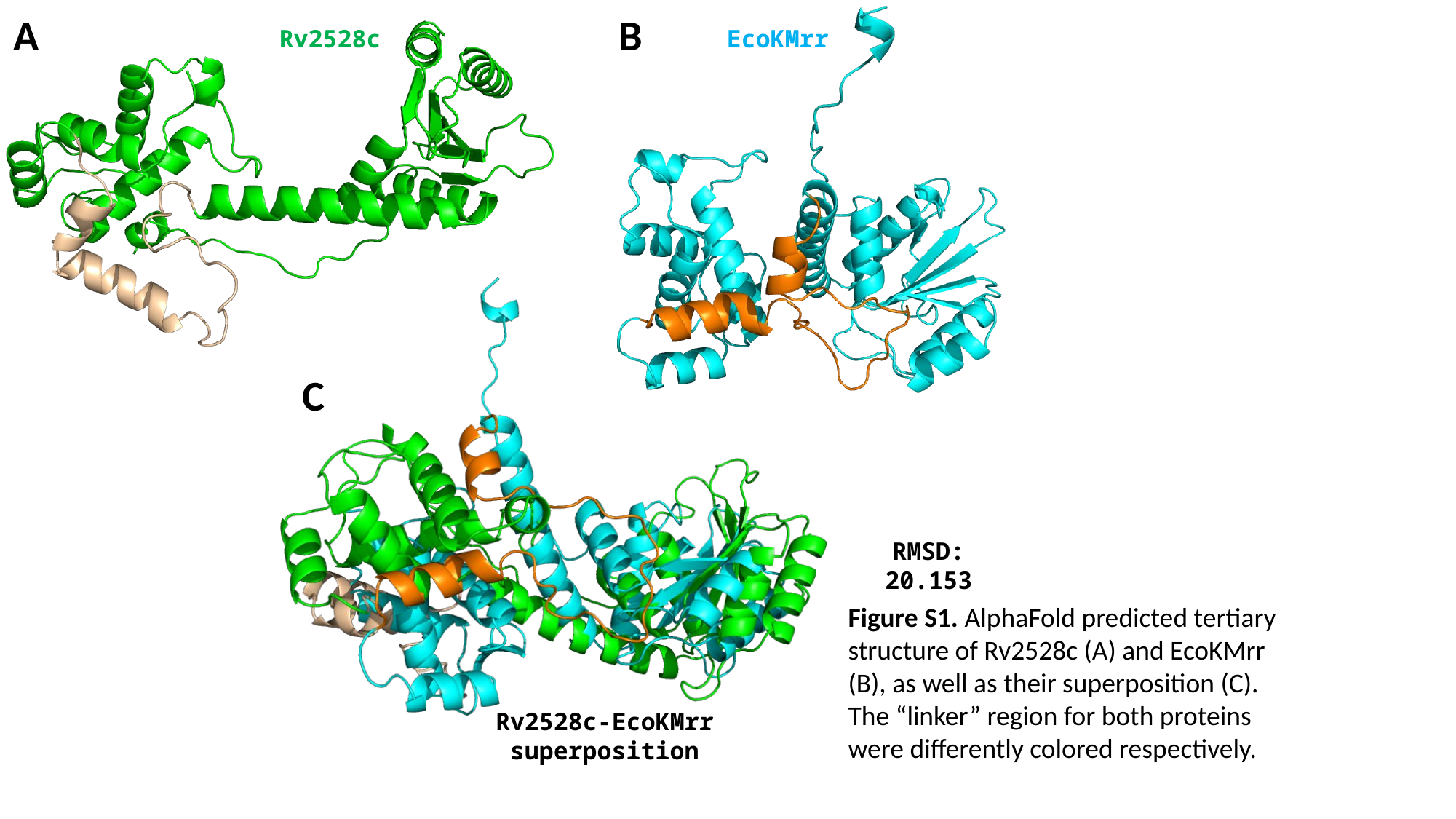

A
B
EcoKMrr
Rv2528c
C
RMSD: 20.153
Figure S1. AlphaFold predicted tertiary structure of Rv2528c (A) and EcoKMrr (B), as well as their superposition (C). The “linker” region for both proteins were differently colored respectively.
Rv2528c-EcoKMrr superposition

## Slide 2
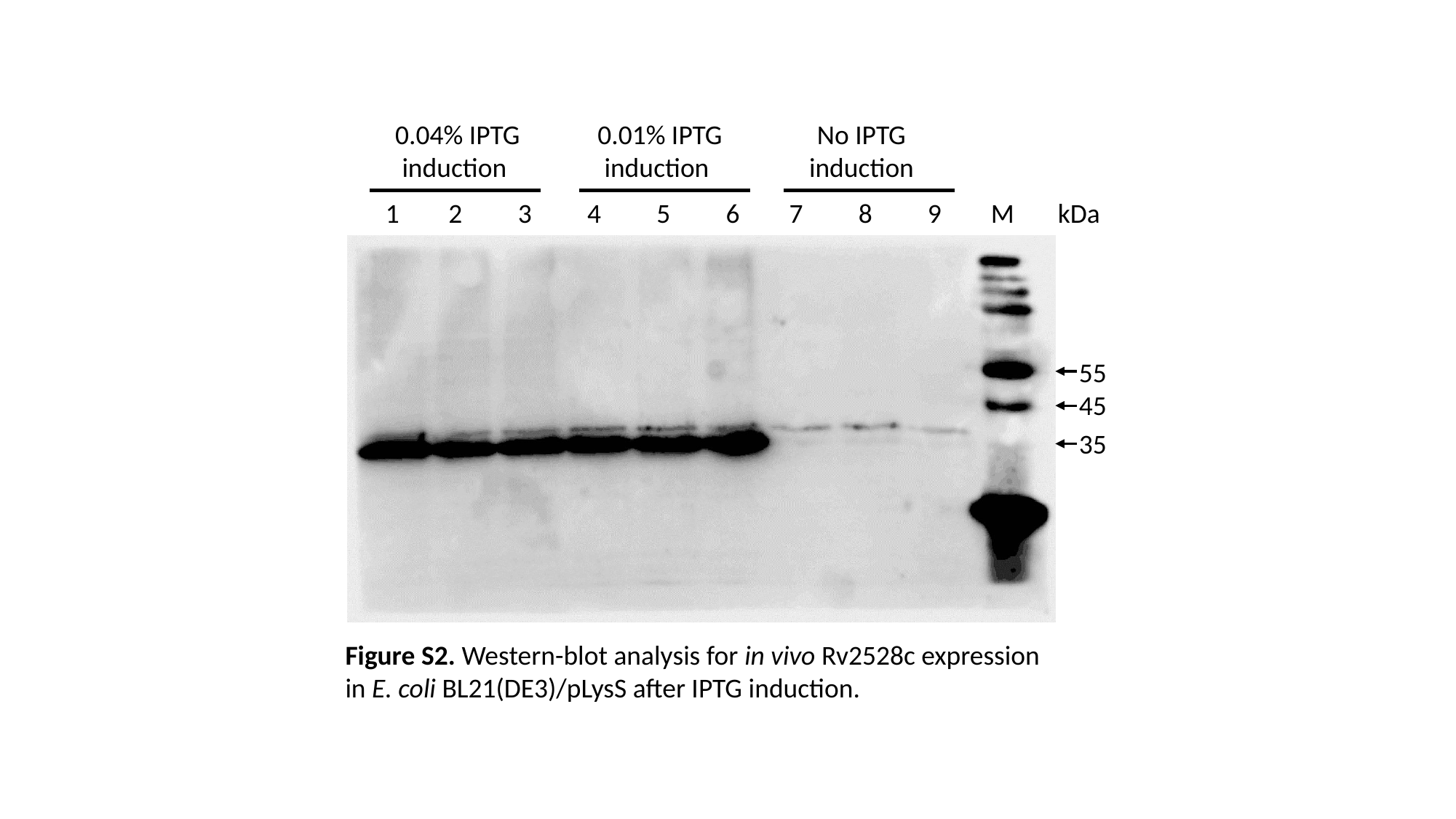

0.04% IPTG
induction
0.01% IPTG
induction
No IPTG
induction
1 2 3 4 5 6 7 8 9 M
kDa
55
45
35
Figure S2. Western-blot analysis for in vivo Rv2528c expression in E. coli BL21(DE3)/pLysS after IPTG induction.

## Slide 3
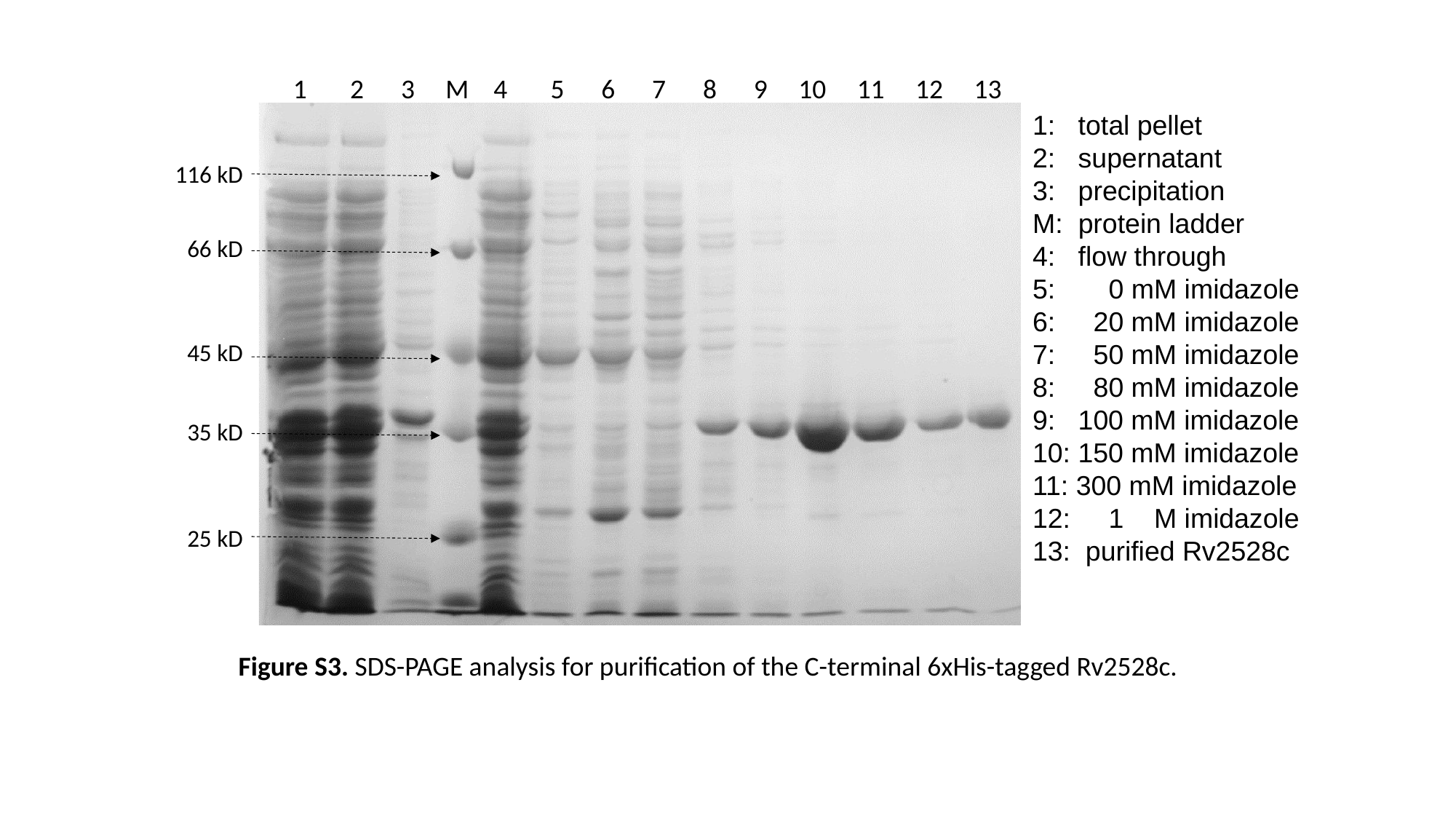

1 2 3 M 4 5 6 7 8 9 10 11 12 13
1: total pellet
2: supernatant
3: precipitation
M: protein ladder
4: flow through
5: 0 mM imidazole
6: 20 mM imidazole
7: 50 mM imidazole
8: 80 mM imidazole
9: 100 mM imidazole
10: 150 mM imidazole
11: 300 mM imidazole
12: 1 M imidazole
13: purified Rv2528c
116 kD
66 kD
45 kD
35 kD
25 kD
Figure S3. SDS-PAGE analysis for purification of the C-terminal 6xHis-tagged Rv2528c.
